# Association of China’s two-child policy with changes in number of births and birth defects rate, 2008–2017

**DOI:** 10.1186/s12889-022-12839-0

**Published:** 2022-03-04

**Authors:** Hanyi Chen, Ting Wei, Haiyin Wang, Yi Zhou, Hua Chen, Lianghong Sun, Shaotan Xiao, Wuren Ma, Huijuan Zhao, Guanghua Chen, Xinlei Liang, Donglan Zhang, Weiwei Zheng, Yixin Zhou, Zhangsheng Yu

**Affiliations:** 1 Science Research and Information Management Department, Pudong New Area Center for Disease Control and Prevention, Shanghai, 200136 China; 2 Fudan University Pudong Institute of Preventive Medicine, Shanghai, China; 3 Department of Bioinformatics and Biostatistics, School of Life Sciences and Biotechnology, Shanghai Jiao Tong University, Shanghai, China; 4 SJTU-Yale Joint Centre for Biostatistics and Data Sciences, Shanghai Jiao Tong University, Shanghai, China; 5 Health Technology Assessment Research Department, Shanghai Health Development Research Centre, Shanghai, 201199 China; 6 Administration office, Pudong New Area Center for Disease Control and Prevention, Shanghai, 200136 China; 7 Key Laboratory of the Public Health Safety, Ministry of Education, Department of Environmental Health, School of Public Health, Fudan University, Shanghai, China; 8 Division of Health Services Research, New York University Long Island School of Medicine, Mineola, USA; 9 Clinical Research Center, Shanghai Jiao Tong University School of Medicine, Shanghai, 201199 China

**Keywords:** Two-child policy, Birth defects, Birth outcomes, Number of births, Difference-in-difference model

## Abstract

**Background:**

In October 2015, China’s one-child policy was universally replaced by a so-called two-child policy. This study investigated the association between the enactment of the new policy and changes in the number of births, and health-related birth outcomes.

**Methods:**

We used difference-in-difference model to analyse the birth record data in Pudong New Area, Shanghai.The design is descriptive before-and-after comparative study.

**Results:**

The data covered three policy periods: the one-child policy period (January 2008 to November 2014); the partial two-child policy period (December 2014 to June 2016); the universal two-child policy period (July 2016 to December 2017). There was an estimate of 7656 additional births during the 18 months of the implementation of the universal two-child policy. The trend of monthly percentage of births to mothers aged ≥35 increased by 0.24 percentage points (95% confidence interval 0.19 to 0.28, *p* < 0.001) during the same period. Being a baby boy, preterm birth, low birth weight, parents with lower educational attainment, and assisted delivery were associated with a higher risk of birth defects.

**Conclusions:**

The universal two-child policy was associated with an increase in the number of births and maternal age. Preterm birth, low birth weight, and assisted delivery were associated with a higher risk of birth defects, which suggested that these infants needed additional attention in the future.

**Supplementary Information:**

The online version contains supplementary material available at 10.1186/s12889-022-12839-0.

## Introduction

The one-child policy, introduced in 1979 by the Chinese Government, was enacted to alleviate population growth, and improve the quality of living for households who cannot afford to have more children [1]. After 30 years, the population ageing, labor shortages, imbalance of sex ratio at birth, and the pressure on social welfare programs suggested that the relaxation of China’s strict one-child policy must be implemented for the country’s development and prosperity [1, 2]. Subsequently, since November 2013, a partial two-child policy was implemented as a transition from the one-child policy to a universal two-child policy, which allowed couples to have two children if either husband or wife was an only-child. This policy was implemented in March 2014 in Shanghai [3]. The universal two-child policy was enacted in October 2015, which allowed all families to have two children.

The impacts of China’s birth policy would be twofold. Firstly, the effects on fertility rate. The multiparous births rate after the enactment of the two-birth policy was an important factor affecting population growth. The estimated number of extra births under the two-child policy would be over 1 million every year [4]. Secondly, the effects on birth outcomes, such as the sex ratio, [5] the proportion of births with advanced maternal age, [6] preterm births, [7, 8] and caesarean delivery, [9, 10] which may also link with a higher prevalence of birth defects (or congenital anomalies) [6, 11].

The Pudong New Area was located in eastern area of Shanghai with a population of 5.5 million, covering 20.89% of the entire city [12]. The primary aim of this paper was to estimate the extra births due to China’s birth control policy change (the partial two-child policy and the universal two-child policy), and to investigate the effects of policy change on birth outcomes. In addition, we aimed to analyze changes in the prevalence of birth defects by types and related risk factors.

## Methods

### Data collection and cleaning

We used hospital-based birth records in Pudong New Area, Shanghai. From January 1, 2008 to December 31, 2017, 428,857 births were reported in the Pudong New Area, Shanghai. Birth information included two parts: birth outcomes (infant gender, birth defect, birth weight, gestational week, household registration, and delivery mode (vaginal delivery or assisted delivery)) and maternal/paternal characteristics (maternal age, paternal age, mother’s education, father’s education, parity, and gravidity). We excluded 6820 (1.59%) birth records with missing individual information and incorrect data (the gestational week is 0, 3, 99 weeks and the age of father/mother is 0, 1, 2, 3, 99 years old). Finally, a total of 422,037 births were analyzed. This study was approved by Institutional Review Board of School of Public Health, Fudan University (IRB00002408 & FWA00002399).

### Three policy periods

The partial two-child policy in Shanghai was implemented in March 2014 and the universal two-child policy was announced in October 2015 [3]. The partial and universal two-child policies were assumed to take effect about 9 months after the announcement of the policy. That is, there was a nine-month lag in the effect of the two-child policy. To better reflect the dynamics of the number of births and policy interventions (the partial two-child policy and the universal two-child policy), three periods were classified based on the time of policy implementation. The three periods were: (1) the one-child policy period from January 2008 to November 2014; (2) the partial two-child policy period from December 2014 to June 2016; and (3) the universal two-child policy period from July 2016 to December 2017.

### Analysis

#### The impact of China’s two-child policy on the number of births

We used the difference-in-difference (DID) model to evaluate the changes in number of births associated with China’s two-child policy [13, 14]. The DID model assessed if the interaction term between time and parity (births to nulliparous or multiparous mothers) was significant or not in the regression model. The model had two assumptions: (1) The births to nulliparous mothers were not affected by the two-child policy and were treated as the control group; (2) The trends of the births to nulliparous mothers and the births to multiparous mothers were parallel during the one-child policy period. The parallel trends would continue if the two-child policy was not implemented. The advantage of this model was that it could adjust for some covariates that were possibly associated with the number of births.

The model was: *Y*_*t*_ = *β*_0_ + *β*_1_ × *policy*_*t*_ + *β*_2_ × *parity*_*t*_ + *β*_3_ × *policy*_*t*_ × *parity*_*t*_ + *β*_4_ × *month dummy*_*t*_ + *e*_*t*_. Where *Y*_*t*_ represented the number of births in month *t*. *policy* was a dummy variable indicating the one-child policy period, the partial two-child policy period, and the universal two-child policy period. *parity* was a dummy variable indicating births to nulliparous or multiparous mothers. There was an association between policy and parity if the interaction term between policy and parity (*β*_3_) in the regression model was not equal to zero. *month dummy* was a dummy variable representing the 12 months in a year.

#### The impact of China’s two-child policy on birth outcomes

We further investigated whether there was an association between China’s two-child policy and birth outcomes. We could not find a control group for which the assumptions of the DID model required, so we used the segmented regression model as a substitute. Segmented regression analysis of the interrupted time series was a powerful statistical method to assess the impact of intervention (China’s two-child policy) on birth outcomes and maternal/paternal characteristics [15–17].

The model was: *Y*_*t*_ = *β*_0_ + *β*_1_ × *month*_*t*_ + *β*_2_ × *partial two child policy*_*t*_ + *β*_3_ × *time after partial two child policy*_*t*_ + *β*_4_ × *unniversal two child policy*_*t*_ + *β*_5_ × *time after universal two child policy*_*t*_ + *e*_*t*_. Here, *Y*_*t*_ was the monthly percentage of birth outcomes; *month* was a continuous variable of time in months (coded as 1 to 120, from January 2008 to December 2017). We used two parameters (level and trend) to define each policy period. The level was the value of birth outcomes right after the policy took effect and the trend was the rate of change or the slope of the regression. *β*_0_ estimated the baseline level during the one-child policy; *β*_1_ estimated the monthly change during the one-child policy (the baseline trend); *β*_2_ estimated the level change immediately after the partial two-child policy took effect; *β*_3_ estimated the change in the trend during the partial two-child policy, compared with the monthly trend of the one-child policy. The sum of the *β*_1_ and *β*_3_ was the slope of the partial two-child policy. *β*_4_ and *β*_5_ estimated the level and the trend of the universal two-child policy, respectively.

#### Risk factors associated with birth defects

Birth defects were diagnosed in the hospital by clinical diagnostic results, genetics, and pathology. Birth defects were reported at birth, and there may be missing report if birth defect was found after a period of times of birth. The birth defect may be under-reporting. Previous studies reported that some risk factors, such as lower educational attainment, low birth weight, and assisted reproductive technology, were associated with higher risks of birth defects [18–20]. We further investigated other possible risk factors associated with birth defects. We initially included all covariates into the logistic regression model and performed variable selection with the best subset selection approach [21]. Candidate variables in the logistic regression model included infant gender (female vs. male), year, maternal age, parity (1 vs. ≥ 2), birth weight (< 2500 g vs. ≥ 2500 g), gestational week (< 37 weeks vs. ≥ 37 weeks), mother’s educational attainment (highly educated mother (college degree or above) vs. others), father’s educational attainment (highly educated father (college degree or above) vs. others), delivery mode (vaginal delivery vs. assisted delivery), household registration (births with Shanghai household registration vs. others). The best subset selection was performed using *regsubsets* () function of *leaps* library for the R version 3.6.3.

#### The prevalence of birth defects subtypes

Birth defects were classified according to the International Classification of Diseases, 10th Revision (ICD10). The specific codes for congenital malformations, deformations, and chromosomal abnormalities were Q00-Q99, which were further subdivided into Q00-Q07 (congenital malformations of the nervous system), Q10–Q18 (congenital malformations of eye, ear, face and neck), Q20–Q28 (congenital malformations of the circulatory system), Q30-Q34 (congenital malformations of the respiratory system), Q35-Q37 (cleft lip and cleft palate), Q38-Q45 (other congenital malformations of the digestive system), Q50-Q56 (congenital malformations of genital organs), Q60-Q64 (congenital malformations of the urinary system), Q65-Q79 (congenital malformations and deformations of the musculoskeletal system); Q90–Q99 (chromosomal abnormalities).

### Patient and public involvement statement

Our investigation is based on the database of the birth records in Pudong New Area, Shanghai. Our research is secondary data analysis which is not the original clinical trial (such as randomised controlled trials) or epidemiology study. Therefore, no patients were involved in the study design, recruitment, and analysis. The results will be disseminated to public, society and government.

## Results

### Trends of monthly births

A total of 422,037 births in the Pudong New Area, Shanghai (an average of 3617 births per month) were included in the analysis. There were 269,101 (3242 births per month on average), 73,629 (3875 births per month on average), and 79,307 (4406 births per month on average) births in the one-child policy period, partial two-child policy period, and universal two-child policy period, respectively.

**Fig. 1:**
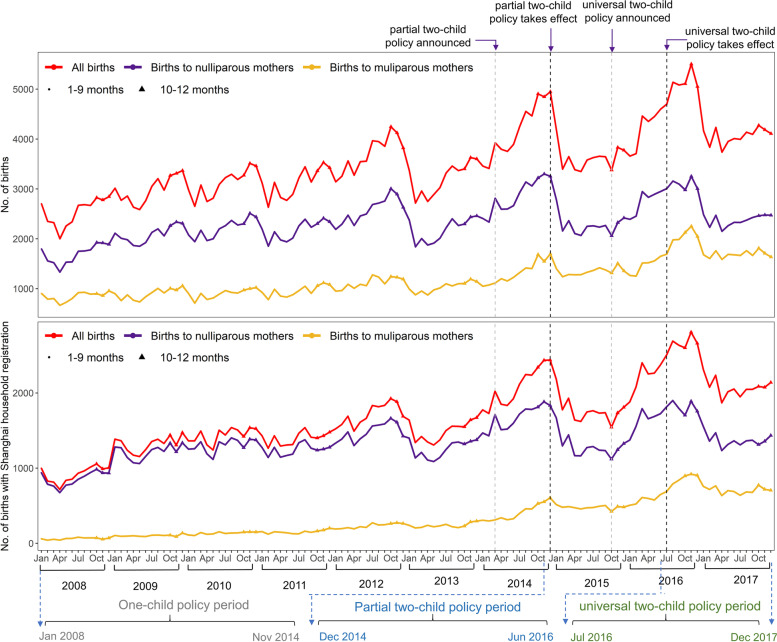
The number of monthly births in Pudong New Area, Shanghai. There were three policy periods: the one-child policy period (Jan 2008 - Nov 2014), the partial two-child policy period (Dec 2014 - Jun 2016), and the universal two-child policy period (Jul 2016 - Dec 2017). Policy was assumed to take effect about nine months after the announcement

The curve according to the number of monthly births and China’s two-child policy interventions was shown in (Fig. 1). Births with non-Shanghai household registration mostly referred to as migrant population. Peaks were seen in the winters of 2014 following the announcement of the partial two-child policy and in 2016 following the announcement of the universal two-child policy. The trends in total monthly births showed birth seasonality, with a birth peak in the winter from October to December. Birth rates were relatively high during the year of the Horse from January 31, 2014 to February 18, 2015 and were relatively low during the year of the Sheep from February 19, 2015 to February 7, 2016.

### Changes in number of births during China’s two-child policy

As shown in Fig. 1, the births to nulliparous mothers and multiparous mothers were nearly parallel during the one-child policy period (the baseline period). This satisfied the parallel trends assumption of the DID model. The regression results showed that there was a significant interaction between China’s two-child policy and parity (Table 1), indicating that the slopes of the regression lines between the number of births to nulliparous mothers and multiparous mothers were different for the different policy periods. The coefficient of the interaction (*β*_3_) indicated how different those slopes were.

**Table 1:** The results of the difference-in-difference model

	Births with Shanghai and non-Shanghai household registration			Births with Shanghai household registration		
	Estimate	Std. Error	***P***-value	Estimate	Std. Error	***P***-value
**Intercept**	2184.94	67.49	2.00E-16	1286.04	44.94	2.00E-16
**partial two-child policy**	261.08	71.58	**3**.**30E-04**	150.58	47.66	**1**.**80E-03**
**universal two-child policy**	331.20	73.20	**9**.**85E-06**	222.63	48.75	**8**.**18E-06**
**parity**	− 1238.40	43.54	**2**.**00E-16**	− 1098.55	28.99	**2**.**00E-16**
**parity∗partial two-child policy**	164.56	100.88	0.10	189.82	67.18	**5**.**15E-03**
**parity∗universal two-child policy**	425.34	103.13	**5**.**25E-05**	331.50	68.68	**2**.**57E-06**
**Jan**	0	–	–	0	–	–
**Feb**	− 152.45	88.69	0.09	−54.80	59.06	0.35
**Mar**	20.25	88.69	0.82	9.45	59.06	0.87
**Apr**	− 113.70	88.69	0.20	−77.25	59.06	0.19
**May**	−70.25	88.69	0.43	−64.95	59.06	0.27
**Jun**	−1.05	88.69	0.99	−22.25	59.06	0.71
**Jul**	92.70	88.94	0.30	4.11	59.23	0.94
**Aug**	167.20	88.94	0.06	36.96	59.23	0.53
**Sep**	115.10	88.94	0.20	26.51	59.23	0.65
**Oct**	193.05	88.94	**3**.**10E-02**	29.31	59.23	0.62
**Nov**	253.60	88.94	**4**.**76E-03**	53.06	59.23	0.37
**Dec**	177.16	88.85	**4**.**74E-02**	30.91	59.16	0.60

#### Births with Shanghai and non-Shanghai household registration

The DID analysis showed that there was an estimate of 425 additional monthly births (95% confidence interval (CI): 322 to 528) during the universal two-child policy period, corresponding to a total of 7656 (95% CI: 5799 to 9512) births during the 18 months of the universal two-child policy period. There was no interaction between the partial two-child policy and parity. The number of births showed a marked seasonality, as there were more births in the winter.

#### Births with Shanghai household registration

There is an interaction between the two-child policy period (partial and universal) and parity for births with Shanghai household registration. There was an estimate of 189 additional monthly births (95% CI: 122 to 257) during the partial two-child policy period and 331 additional monthly births (95% CI: 262 to 400) during the universal two-child policy period. Comparing with those in the partial two-child policy period, there were 142 additional monthly births during the universal two-child policy period.

**Fig. 2:**
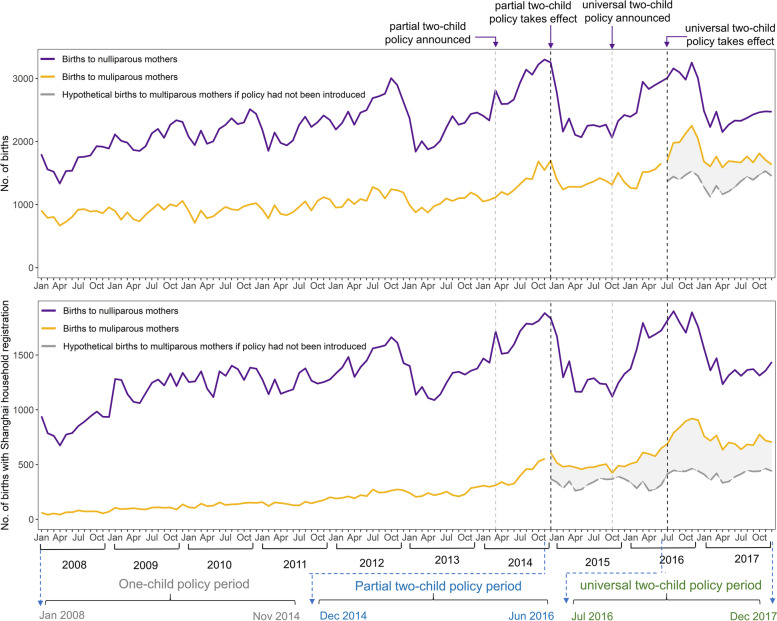
The estimated number of monthly births if the two-child policy had not been implemented. The purple line was the monthly number of nulliparous births; the yellow line was the monthly number of multiparous birth; the grey dashed line was the estimated number of multiparous birth if the two-child policy had not been implemented (For births with Shanghai and non-Shanghai household registration, the coefficients of parity*universal two-child policy was zero in the difference-in-difference model; For births with Shanghai household registration, the coefficients of parity*partial two-child policy and parity*universal two-child policy were zero); The area of the grey shading was estimated extra births during to China’s policy change

Hypothetical births to multiparous mothers were calculated using the coefficients of the DID model if the policy had not been implemented (Fig. 2).

### Changes in birth outcomes during China’s two-child policy

**Fig. 3:**
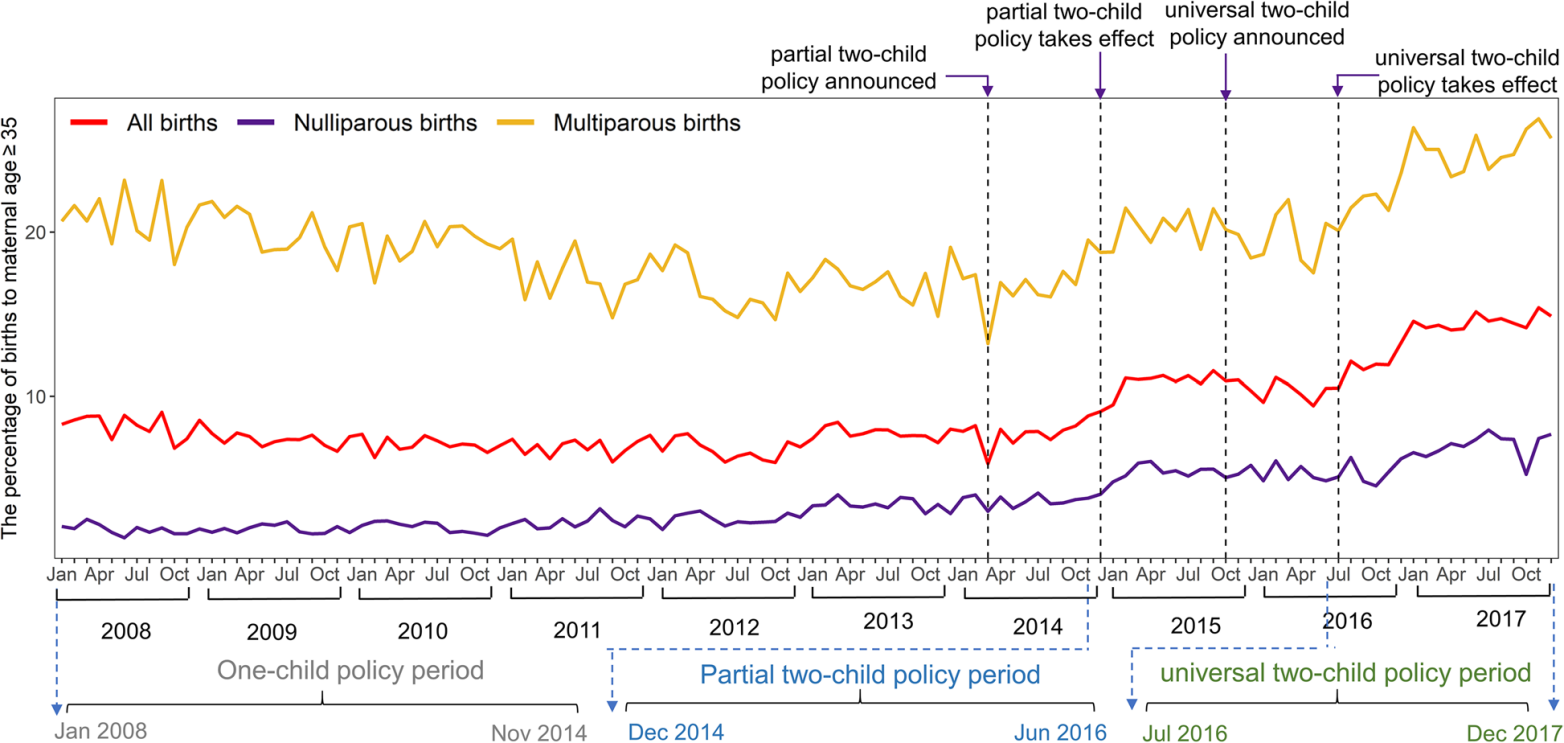
The monthly percentage of births to maternal age ≥ 35

We plotted the monthly percentage of births to mothers aged ≥35 (Fig. 3). It noticeably increased from 7.39% during the one-child policy period, to 10.59% during the partial two-child policy period, and to 13.65% during the universal two-child policy period (Table 2). We used the segmented regression model of interrupted time series to access if the change of the monthly percentage was significantly related to the policy. The estimated monthly percentage of births to mothers aged ≥35 rose abruptly by 3.42% (95% CI: 3.04 to 3.81, *p* < 0.001) immediately after the partial two-child policy took effect. The percentage increased again both in level (*β*_4_ = 0.97, 95% CI: 0.49 to 1.46, *p* < 0.05) and in trend (*β*_5_= 0.24, 95% CI: 0.19 to 0.28, *p* < 0.001) during the universal two-child policy. There was a significantly higher percentage in multiparous births with maternal ≥35 than nulliparous births with maternal ≥35 (*p* < 0.001). The Pearson correlation coefficient between mother’s age and father’s age was 0.77, and they were highly associated. Similar pattern was observed for births to paternal age ≥ 35 (S1 Figure).

**Table 2:** Changes in birth characteristics during China’s two-child policy

		Monthly percentage % (mean (standard deviation))			The partial two-child policy period		The universal two-child policy period	
		one-child policy period	partial two-child policy period	universal two-child policy period	Level change *β*_2_ (95% CI, *P*-value)	Trend change *β*_3_ (95% CI, *P*-value)	Level change *β*_4_ (95% CI, *P*-value)	Trend change *β*_5_ (95% CI, *P*-value)
**Maternal characteristics**	**mother age ≥ 35**	7.39 (0.72)	10.59 (0.72)	13.65 (1.42)	3.42 (3.04 to 3.81, < 0.001)	−0.01 (− 0.04 to 0.03, 0.861)	0.97 (0.49 to 1.46, 0.045)	0.24 (0.19 to 0.28, < 0.001)
	Nulliparous births	2.51 (0.72)	5.29 (0.51)	6.46 (1.06)	1.64 (1.38 to 1.9, < 0.001)	−0.01 (− 0.03 to 0.01, 0.513)	−0.25 (− 0.58 to 0.07, 0.438)	0.13 (0.1 to 0.16, < 0.001)
	Multiparous births	18.27 (2.13)	19.89 (1.29)	24.03 (1.95)	4.5 (3.75 to 5.24, < 0.001)	0.04 (−0.02 to 0.1, 0.513)	1.61 (0.68 to 2.55, 0.087)	0.31 (0.23 to 0.4, < 0.001)
**Birth outcomes**	**Caesarean delivery**	54.02 (3.58)	47.86 (1.62)	47.73 (1.45)	−3.84 (−5.44 to −2.25, 0.018)	−0.03 (− 0.16 to 0.09, 0.786)	−0.81 (− 2.81 to 1.2, 0.688)	0.21 (0.03 to 0.4, 0.254)
	Nulliparous births	57.19 (5.19)	44.51 (2.12)	43.73 (1.58)	−5.77 (−7.64 to −3.9, 0.003)	−0.01 (− 0.16 to 0.14, 0.97)	−0.97 (− 3.32 to 1.39, 0.683)	0.29 (0.07 to 0.51, 0.186)
	Multiparous births	46.88 (4.68)	53.82 (1.8)	53.52 (1.7)	−0.55 (−1.71 to 0.61, 0.634)	− 0.11 (− 0.2 to − 0.01, 0.253)	−1.65 (− 3.11 to − 0.19, 0.26)	0.02 (− 0.11 to 0.16, 0.861)
	**Sex ratio**	114.32 (4.33)	110.44 (3.22)	109.86 (3.81)	−0.86 (− 1.31 to − 0.4, 0.062)	0.06 (0.02 to 0.1, 0.11)	−0.06 (− 0.64 to 0.51, 0.911)	−0.1 (− 0.15 to − 0.05, 0.065)
	Nulliparous births	107.61 (4.51)	106.54 (3.75)	106.71 (4.45)	−3.45 (− 5.75 to − 1.15, 0.136)	0.27 (0.08 to 0.45, 0.149)	0.29 (− 2.61 to 3.18, 0.922)	− 0.52 (− 0.79 to − 0.25, 0.056)
	Multiparous births	131.51 (10.48)	117.78 (6.47)	114.61 (5.79)	−7.63 (− 12.26 to − 3.01, 0.102)	0.28 (− 0.09 to 0.65, 0.453)	−2.26 (− 8.08 to 3.56, 0.699)	− 0.3 (− 0.85 to 0.24, 0.582)
	**Gestation < 37 weeks**	5.22 (0.97)	7.77 (0.66)	7.84 (0.73)	0.69 (0.35 to 1.02, 0.041)	0.03 (0 to 0.06, 0.241)	−1.15 (− 1.56 to − 0.73, 0.007)	0.01 (− 0.03 to 0.05, 0.845)
	Nulliparous births	4.55 (0.85)	6.67 (0.73)	6.85 (0.71)	0.41 (0.08 to 0.74, 0.216)	0.05 (0.02 to 0.08, 0.058)	−1.17 (− 1.58 to − 0.75, 0.006)	0 (− 0.04 to 0.04, 0.918)
	Multiparous births	6.72 (1.49)	9.71 (0.93)	9.27 (0.97)	0.72 (0.19 to 1.25, 0.174)	0 (− 0.04 to 0.04, 0.962)	−1.38 (−2.05 to − 0.72, 0.04)	0.02 (− 0.05 to 0.08, 0.804)
	**Birth weight < 2500 g**	3.67 (0.56)	5.3 (0.54)	5.36 (0.43)	0.53 (0.3 to 0.77, 0.024)	0.04 (0.02 to 0.06, 0.036)	−0.83 (−1.12 to − 0.53, 0.006)	−0.01 (− 0.04 to 0.02, 0.706)
	Nulliparous births	3.22 (0.52)	4.72 (0.68)	4.86 (0.35)	0.41 (0.17 to 0.65, 0.092)	0.05 (0.03 to 0.07, 0.008)	−0.67 (− 0.97 to − 0.36, 0.031)	−0.04 (− 0.07 to − 0.01, 0.182)
	Multiparous births	4.68 (0.96)	6.34 (0.62)	6.09 (0.73)	0.48 (0.07 to 0.9, 0.249)	0.02 (−0.01 to 0.05, 0.572)	−1.2 (− 1.72 to − 0.68, 0.023)	0.03 (− 0.02 to 0.07, 0.605)
	**Birth defect**	0.98 (0.19)	1.17 (0.3)	0.82 (0.15)	0.28 (0.18 to 0.39, 0.009)	−0.01 (− 0.02 to 0, 0.386)	−0.17 (− 0.3 to − 0.03, 0.216)	0 (−0.02 to 0.01, 0.755)
	Nulliparous births	0.96 (0.23)	1.12 (0.31)	0.76 (0.18)	0.34 (0.22 to 0.47, 0.007)	−0.01 (− 0.02 to 0, 0.312)	−0.17 (− 0.33 to − 0.02, 0.271)	0 (−0.01 to 0.02, 0.874)
	Multiparous births	1.02 (0.34)	1.24 (0.41)	0.9 (0.22)	0.14 (−0.03 to 0.32, 0.42)	0 (−0.02 to 0.01, 0.893)	− 0.18 (− 0.4 to 0.04, 0.426)	−0.02 (− 0.04 to 0, 0.407)

During the one-child policy period, the trend of the monthly percentage of caesarean delivery decreased for all births (*β*_1_ = − 0.04, 95% CI: − 0.05 to − 0.02, *p* = 0.006) and for nulliparous births (*β*_1_ = − 0.13, 95% CI: − 0.15 to − 0.12, *p* < 0.001), however, the trend for multiparous births rose over time (*β*_1_ = 0.17, 95% CI: 0.16 to 0.18, *p* < 0.001) (S2 Figure). For nulliparous births, multiparous births, and all births, there was no significant monthly change in the percentage of caesarean delivery during the partial two-child policy and the universal two-child policy periods. The monthly percentage of births to multiparous births was consistently higher than nulliparous births during the partial and universal two-child policy periods.

The sex ratio at birth was defined as the number of male births to every 100 female births, and the natural sex ratio should be around 105 [5]. There was a significantly higher sex ratio to multiparous births (126.8 ± 11.73) than nulliparous births (107.3 ± 4.38) (*p* < 0.001) (S3 Figure). The trend of sex ratio to nulliparous births had no significant change during the one-child (107.61 ± 4.51), the partial two-child (106.54 ± 3.75), and the universal two-child policy periods (106.71 ± 4.45), and were almost at the normal level. The trend of the sex ratio declined for all births (*β*_1_ = − 0.01, 95% CI: − 0.02 to − 0.01, *p* = 0.005) and multiparous births (*β*_1_ = − 0.17, 95% CI: − 0.21 to − 0.13, *p* < 0.001) during the one-child policy period. However, there was no significant change of sex ratio to all births and multiparous births during the partial and universal two-child policy periods.

Preterm birth was less than 37 completed weeks of gestation defined by the World Health Organization [8, 22]. The trend of percentage of preterm birth gradually rose over time for all births (*β*_1_ = 0.03, 95% CI: 0.027 to 0.033, *p* < 0.001), nulliparous births (*β*_1_ = 0.024, 95% CI: 0.021 to 0.026, *p* < 0.001), and multiparous births (*β*_1_ = 0.045, 95% CI: 0.04 to 0.05, *p* < 0.001) during the one-child policy period. There was no significant change in the trend during the partial two-child policy period (*p* = 0.241) and the universal two-child policy period (*p* = 0.845). The monthly percentage of preterm birth to multiparous mothers were constantly higher than nulliparous mothers from 2008 to 2017 (S4 Figure). The similar trend existed for the percentage of low birth weight (< 2500 g), rising from 3.67% during the one-child policy period to 5.3% during the partial two-child policy, and to 5.36% during the universal two-child period (S5 Figure) [23].

The monthly percentage of birth defects increased from 0.98% during the one-child policy period to 1.17% during the partial two-child policy, and decreased to 0.82% during the universal two-child policy (S6 Figure). The estimated percentage of birth defect increased by 0.28% (95% CI: 0.18 to 0.39, *p* = 0.009) immediately after the partial two-child policy took effect. The trends of the percentage of birth defects did not change significantly during the one-child, partial two-child, and universal two-child policy periods. There was no significant difference for the monthly percentage of birth defects between nulliparous births (0.96 ± 0.26) and multiparous births (1.04 ± 0.35) (*p* = 0.09).

### Risk factors associated with birth defects

**Fig. 4:**
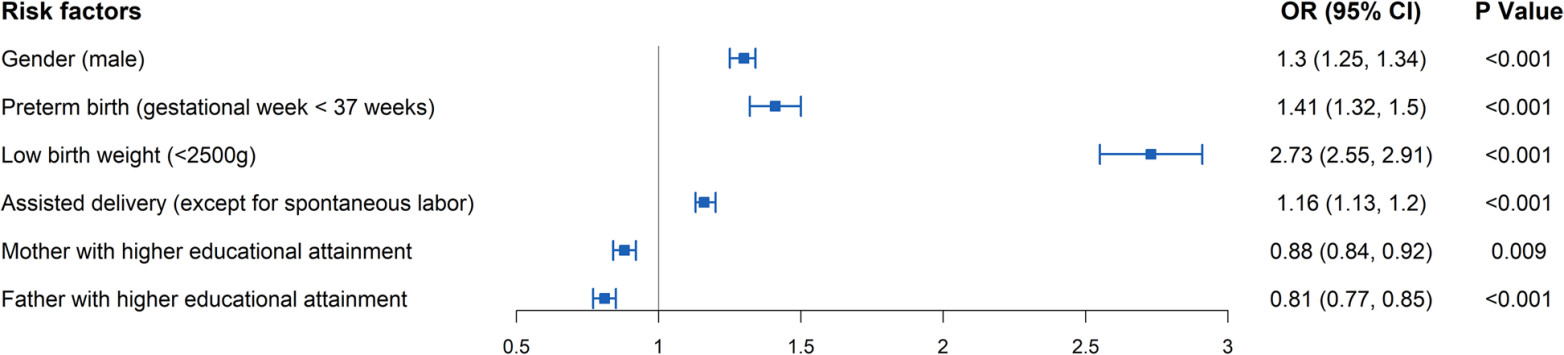
The logistic regression model identifying the risk factors associated with birth defects

We used the adjusted *R*^2^ to select the best subset model. After best subset selection, six variables (baby being male, preterm birth (gestational week < 37 weeks), low birth weight (< 2500 g), father with lower educational attainment, mother with lower educational attainment, and assisted delivery (except for vaginal delivery)) were associated with a higher risk of birth defects (Fig. 4).

## Discussion

### Three primary findings

#### The two-child policy was associated with a moderate increase in fertility

For births with Shanghai and non-Shanghai household registration, we estimated that the universal two-child policy was associated with a total of 7656 (95% CI: 5799 to 9512) incremental number of births during the 18 months of the universal two-child policy period, however, the partial two-child policy was not associated with fertility. For births with Shanghai household registration, there was an estimate of 3607 incremental number of births (95% CI: 2330 to 4883) during the 19 months of the partial two-child policy period and 5967 incremental number of births (95% CI: 4731 to 7203) during the 18 months of the universal two-child policy period. There was only as rise of births with Shanghai household registration during the partial two-child policy period. The partial two-child policy was implemented in different province at different times. So, the results were reasonable.

#### The changes of birth outcomes after the policy change

The partial and universal two-child policies were also significantly related to an increase in births to mothers with maternal age ≥ 35 (from 7.39% during the one-child policy period, to 10.59% during the partial two-child policy period, and to 13.65% during the universal two-child policy). The most noticeable finding was that the trend of monthly percentage of births to parents with maternal ≥35 rose for multiparous births and nulliparous births during the universal two-child policy period, although the change of the monthly percentage for multiparous births was more rapidly than for nulliparous births during the universal two-child policy period.

The monthly percentage of caesarean delivery decreased to nulliparous women and increased to multiparous women during the one-child policy period. This was possible due to the specific policies implemented by the health departments in China to reduce unnecessary caesarean sections, such as setting goals for hospitals to reduce the caesarean section rates [10]. The trend of the monthly percentage of caesarean delivery showed no significant change during the partial and universal two-child policy periods. Compared with nulliparous women, multiparous women exhibited higher caesarean delivery rate, which may be related to the higher rate of repeat caesarean delivery for multiparous women who had caesarean delivery previously [24, 25].

The trend of the monthly percentage of preterm birth and low birth weight increased during the one-child policy and then remained stable during the partial and universal two-child policy periods.

#### The prevalence of birth defects subtypes and risk factors associated with birth defects

The trends of monthly percentage of birth defects did not change significantly during the partial two-child and universal two-child policy periods. This may be due to the enhanced public awareness of prenatal diagnosis that have been developed by the Chinese government to enhance prenatal screening and universal folic acid supplement use [26]. The top three subtypes of birth defects were the musculoskeletal system (Q65-Q79), the circulatory system (Q20-Q28), and the eye, ear, face, and neck system (Q10-Q18) during all periods. The musculoskeletal system problems remained the predominant birth defects during all periods, however, the circulatory system problems sharply increased and then rank the first during the partial two-child policy period (S7 Figure).

The best subset selection showed that six factors (baby being male, preterm birth, low birth weight, father with lower educational attainment, mother with lower educational attainment, and assisted delivery) were associated with a higher risks of birth defects. Previous studies have also reported that low educational attainment, low birth weight, and assisted reproductive technology were associated with higher risks of birth defects [18–20].

Both the percentage of nulliparous births with maternal ≥35 and the percentage of nulliparous births with maternal ≥35 rose during the universal two-child policy. More attention is needed to ensure the health of the increasing number of advanced maternal age. Low birth weight and preterm birth were associated with birth defect, and it was necessary to improve health care and follow-up care for these infants.

### Strengths and limitations

This paper had two main advantages. Firstly, the long period of time series data (from 2008 to 2017) allowed us to study long-term associations among three different policy periods. Secondly, the complete information for births allowed us to systematically estimate the associations between changes in China’s birth control policy and the fertility, birth outcomes, maternal/paternal characteristics, and birth defects. There are some limitations to our study. Firstly, our data only included birth records in Shanghai, which could not be generalized to other areas of China. Secondly, we did not consider the changes in the overall socioeconomic systems and the structure of women of childbearing that may be confounders. We found that the trend of monthly percentage of births with maternal ≥35 rose both for nulliparous births and multiparous birth during the two-child policy period. Researches have showed that many Chinese couples defer childbearing because expense, especially those living in the big city, such as Shanghai [27].

## Conclusions

There was an increasing rate of births with advanced maternal (age ≥ 35). More resources and prenatal screening services were needed to prevent and treat birth defects in the future.

## Supplementary Information

**Additional file 1.**

## Data Availability

The data that support the findings of this study are available from the corresponding author on reasonable request. Participant data without names and identifiers will be made available after approval from the corresponding authors, Shanghai Pudong CDC and Chinese government. After publication of study findings, the data will be available for others to request. The research team will provide an email address for communication once the data are approved to be shared with others. The proposal with detailed description of study objectives and statistical analysis plan will be needed for evaluation of the reasonability to request for our data. The corresponding author Pudong CDC and Chinese government will make a decision based on these materials. Additional materials may also be required during the process.
