# Supplementary material for: Association of China’s two-child policy with changes in number of births and birth defects rate, 2008–2017
Source: BMC Public Health. 2022 Mar 4;22:434. doi: 10.1186/s12889-022-12839-0 (PMC8895506; doi:10.1186/s12889-022-12839-0)
Supplement: Supplementary file 1 — Additional file 1. [file 12889_2022_12839_MOESM1_ESM.docx]

**Association of China’s two-child policy with changes in number of births and birth defects rate, 2008-2017**

**Supplementary Materials**

Hanyi Chen ^1,2,†^, Ting Wei ^3,4,†^, Haiyin Wang ^6,†^, Yi Zhou ^1,2^, Hua Chen ^1,2^, Lianghong Sun ^1,2^, Shaotan Xiao ^2,7^, Wuren Ma ^8^, Huijuan Zhao ^8^, Guanghua Chen ^8^, Xinlei Liang ^8^, Donglan Zhang ^9^, Weiwei Zheng ^8,*^, Yixin Zhou ^2,7,*^, Zhangsheng Yu ^3,4,5,*^

^1^ Science Research and Information Management Department, Pudong New Area Center for Disease Control and Prevention, Shanghai, 200136, China

^2^ Fudan University Pudong Institute of Preventive Medicine, Shanghai, China

^3^ Department of Bioinformatics and Biostatistics, School of Life Sciences and Biotechnology, Shanghai Jiao Tong University, Shanghai, China

^4^ SJTU-Yale Joint Centre for Biostatistics and Data Sciences, Shanghai Jiao Tong University, Shanghai, China

^5^ Clinical Research Center, Shanghai Jiao Tong University School of Medicine, Shanghai, 201199, China

^6^ Health Technology Assessment Research Department, Shanghai Health Development Research Centre, Shanghai, 201199, China

^7^ Administration office, Pudong New Area Center for Disease Control and Prevention, Shanghai, 200136, China

^8^ Key Laboratory of the Public Health Safety, Ministry of Education, Department of Environmental Health, School of Public Health, Fudan University

^9^ Department of Health Policy and Management, College of Public Health, University of Georgia

† These authors contributed equally to the work.

* Corresponding author. E-mail addresses: weiweizheng@fudan.edu.cn (Weiwei Zheng); 18930733119@163.com (Yixin Zhou); yuzhangsheng@sjtu.edu.cn (Zhangsheng Yu).

# Supplementary Results

## Changes in the prevalence of birth defects subtypes

The top three subtypes of congenital malformations and chromosomal abnormalities were Q65-Q79 (the musculoskeletal system), Q20-Q28 (the circulatory system), and Q10-Q18 (eye, ear, face and neck) during all periods. The prevalence of Q65-Q79 (the musculoskeletal system) ranked first across the one-child policy period and universal two-child policy period. The prevalence of Q20-Q28 (the circulatory system) rose and ranked first during the partial two-child policy. The highest prevalence of birth defect subtypes was Q65-Q79 (36·88%), Q20-Q28 (34·56%), and Q65-Q79 (32·04%) during the one-child policy period, the partial two-child policy period, and the universal two-child policy period, respectively (Supplementary Figure 9).

The prevalence of Q20-Q28 (the circulatory system) significantly rose from 14·05 during the one-child policy to 39·66 (OR =2·82, 95% confidence interval 2·42 to 3·31, P<0·001) during the partial two-child policy period. The prevalence then decreased to 20·05 during the universal two-child policy period.

The prevalence of Q00-Q07 (the nervous system), Q10-Q18 (eye, ear, face and neck), and Q65-Q79 (the musculoskeletal system) gradually decreased in the partial two-child policy period and universal two-child policy period. For example, the prevalence of Q10-Q18 significantly decreased from 18·62 during the one-child policy period, to 13·31 during the partial two-child policy period (OR=0·71, 95% confidence interval 0·57 to 0·89, P=0·002), and to 11·1 during the universal two-child policy period (OR =0·6, 95% confidence interval 0·47 to 0·75, P<0·001).

The prevalence of Q35-Q37 (cleft lip and cleft palate) and Q38-Q45 (the digestive system) showed no significant change during the partial two-child policy period, and then decreased during the universal two-child policy period. The prevalence of Q35-Q37 significantly decreased from 10·07 during the one-child policy period, to 5·8 during the universal two-child policy period (OR =0·58, 95% confidence interval 0·41 to 0·79, P<0·001).

In the partial two-child policy period and universal two-child policy period, the prevalence (per 10, 000 births) of Q30-Q34 (the respiratory system), Q50-Q56 (genital organs), Q60-Q64 (the urinary system), and Q90-Q99 (chromosomal abnormalities) showed no significant difference compared with one-child policy.

# 2. Supplementary Figures


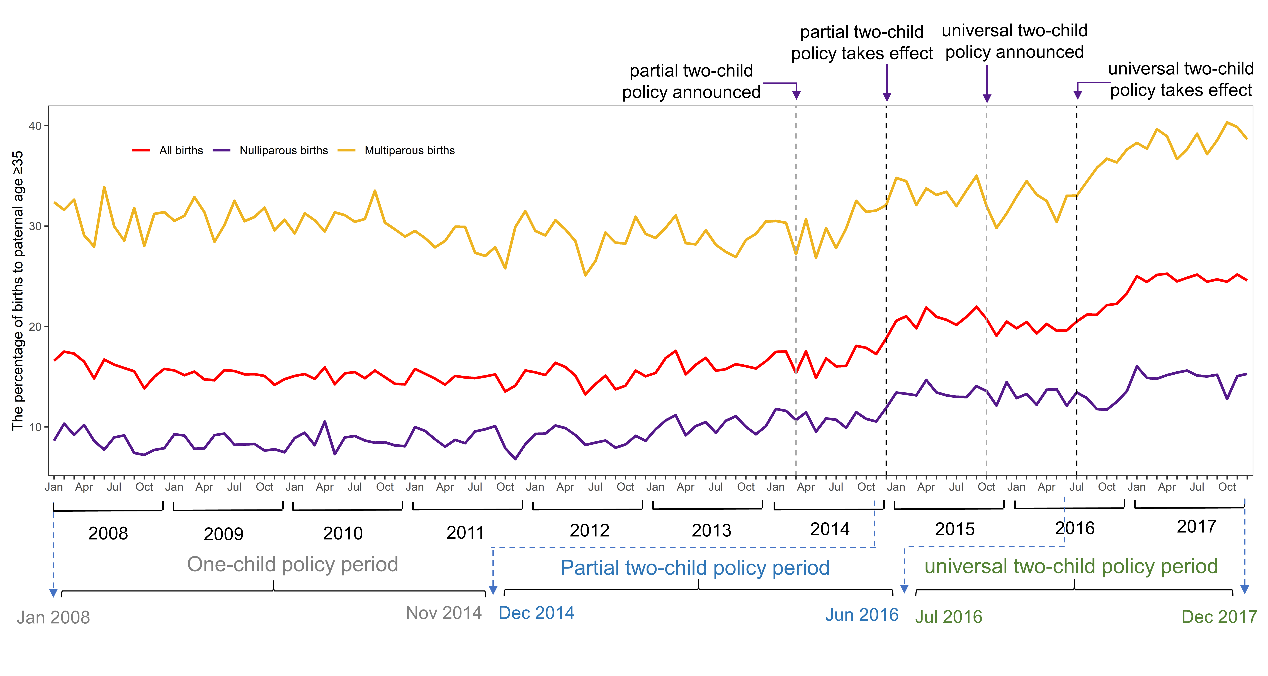


**Supplementary Figure 1. The monthly percentage of births to paternal age ≥ 35.**


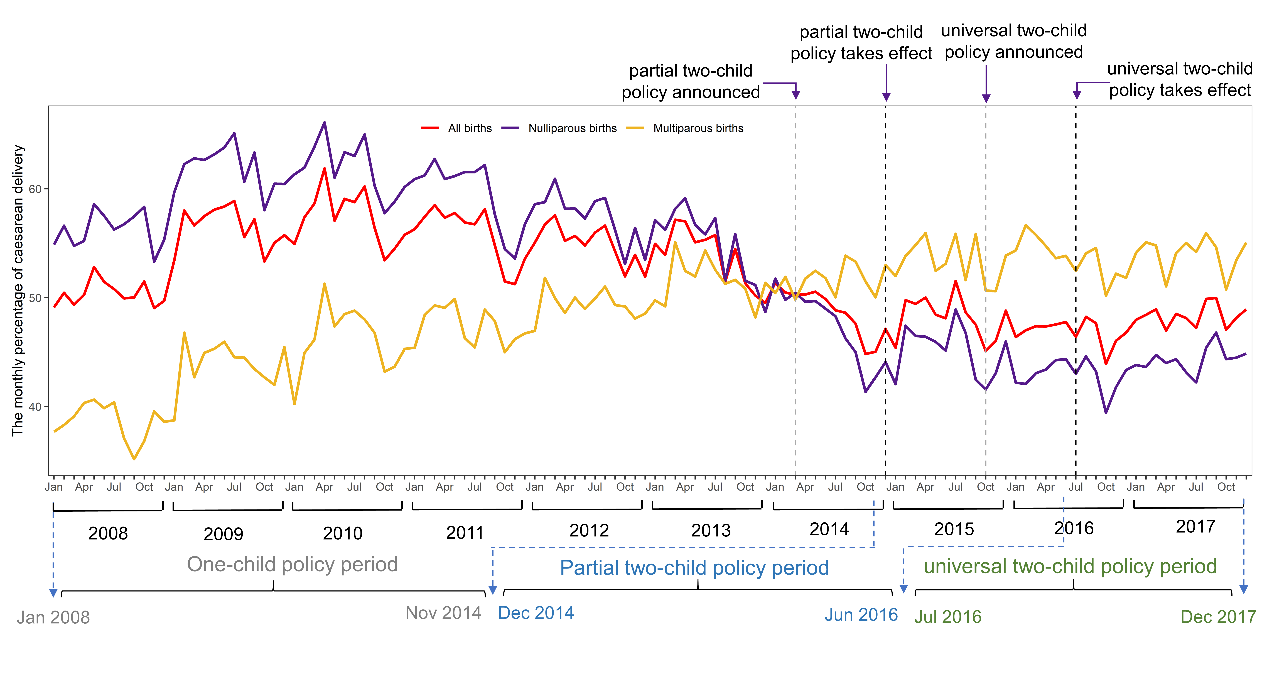


**Supplementary Figure 2. The monthly percentage of caesarean delivery.**


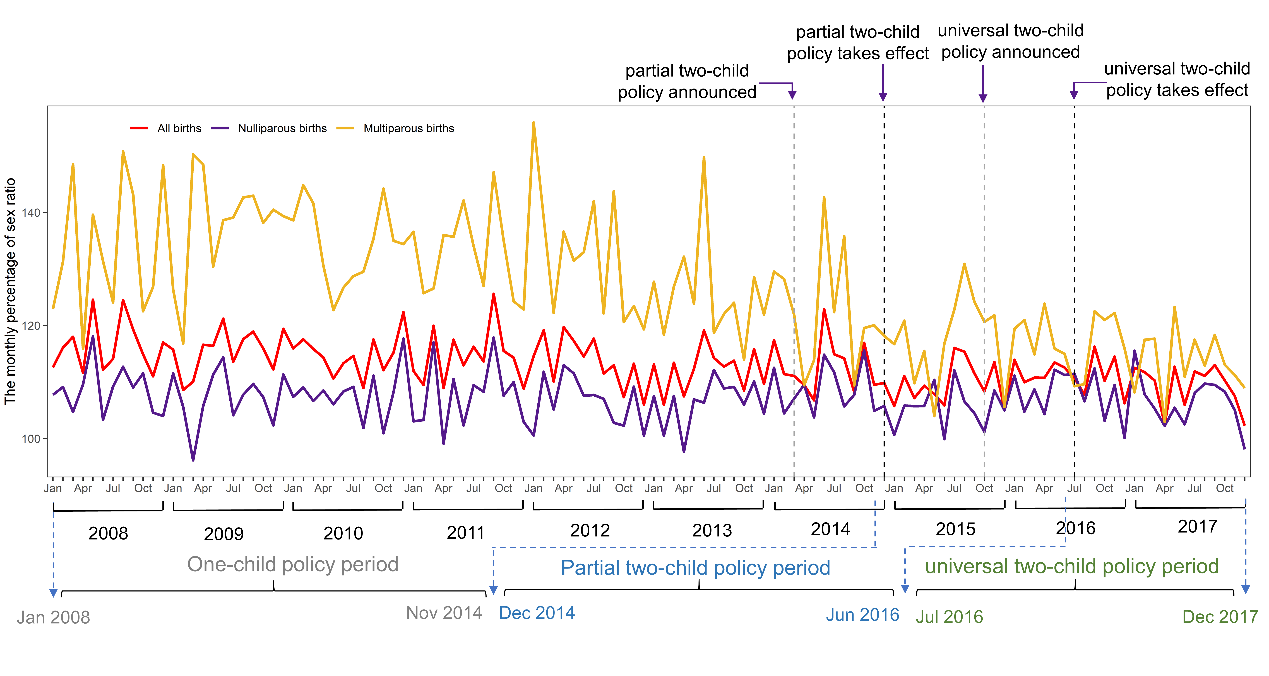


**Supplementary Figure 3. The monthly sex ratio at birth.**


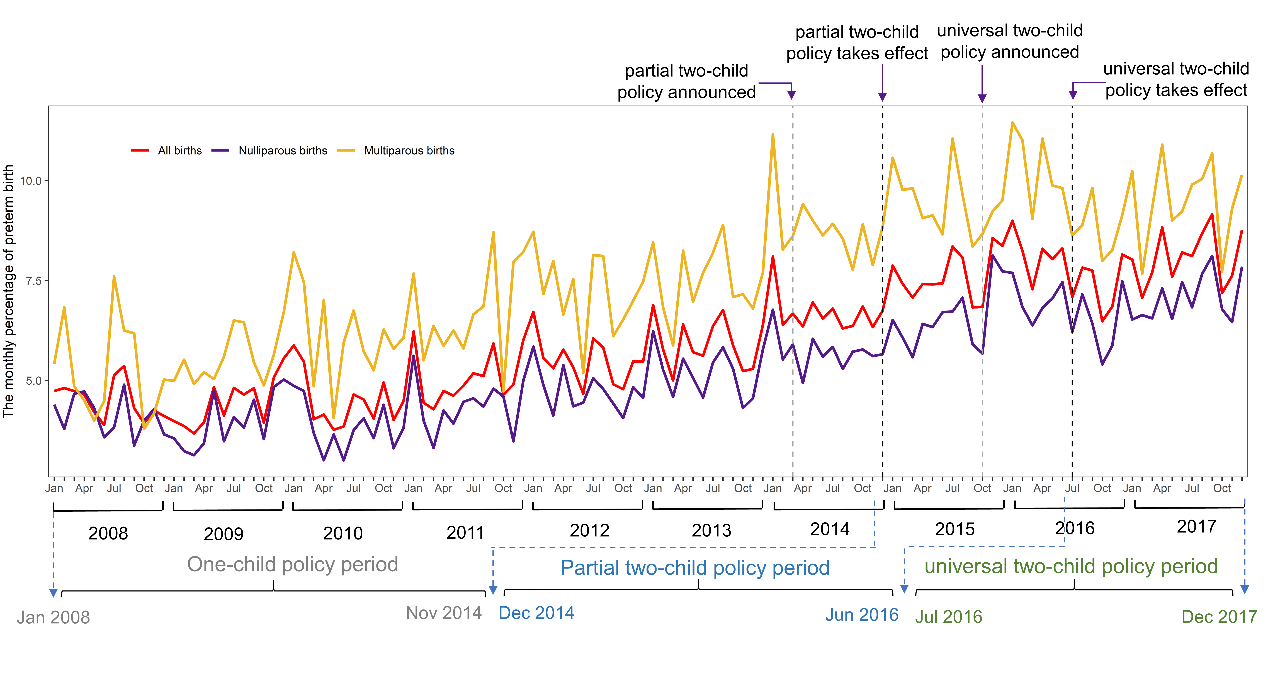


**Supplementary Figure 4. The monthly percentage of preterm birth.**


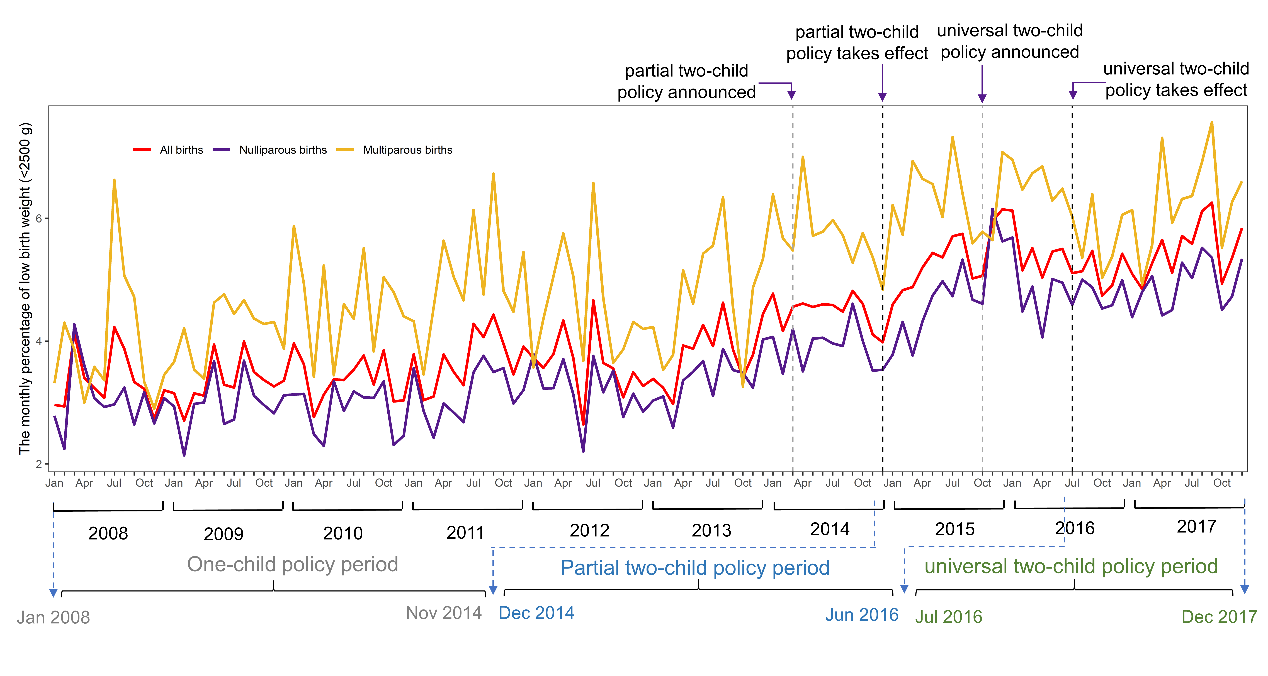


**Supplementary Figure 5. The monthly percentage of low birth weight.**


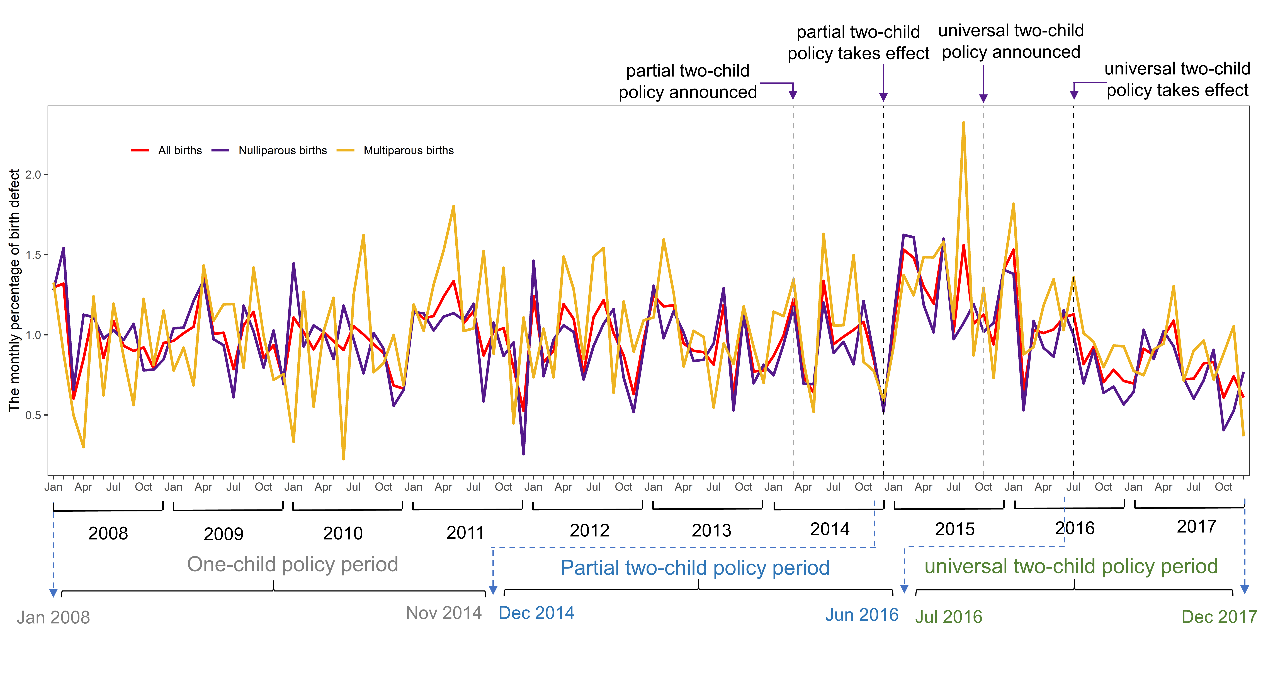


**Supplementary Figure 6. The monthly percentage of birth defects.**


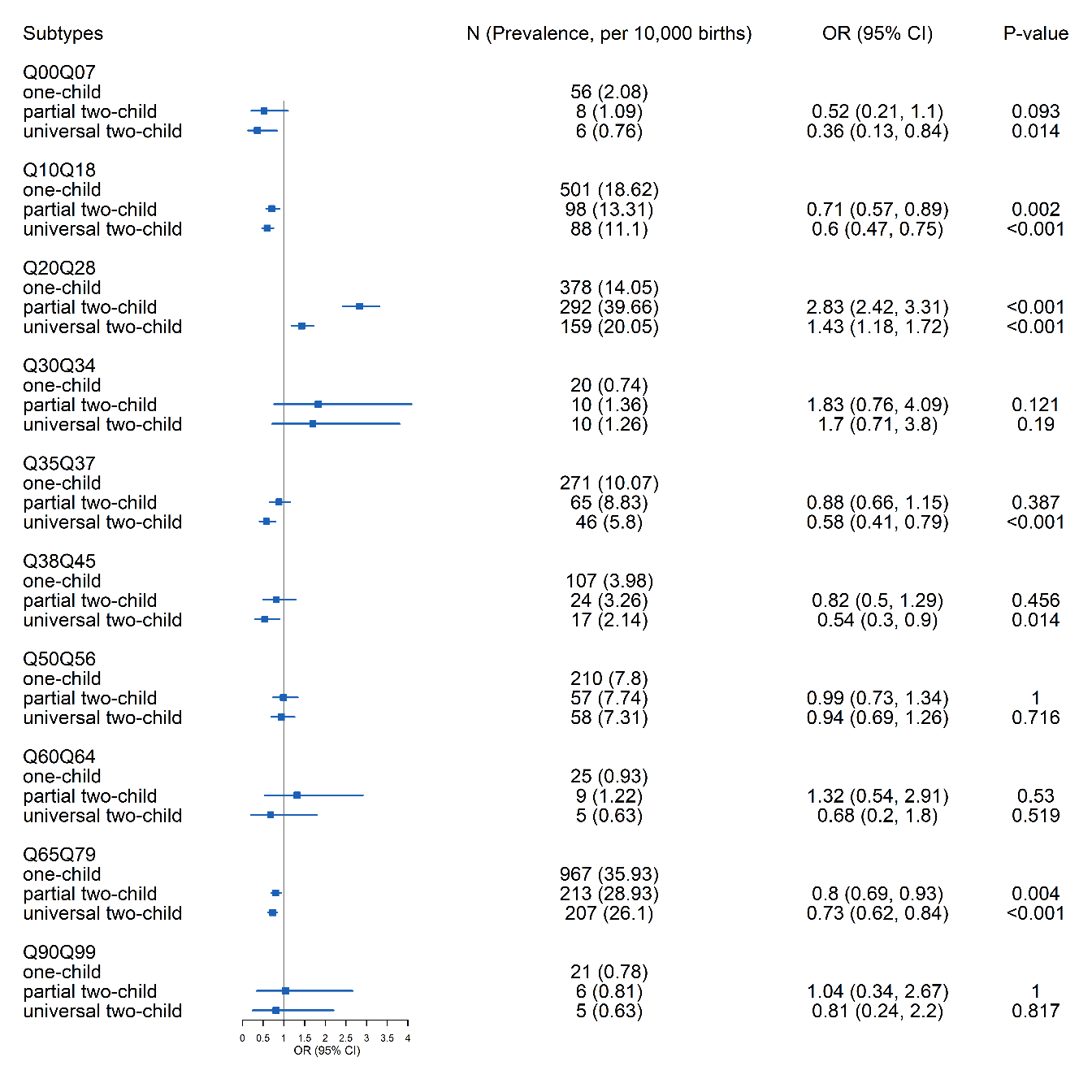


**Supplementary Figure 7. The association between the prevalence of birth defects subtypes and policy periods.** N was the number of birth defects during the corresponding policy period. The one-child policy period was as baseline. OR, 95% confidence interval, and P-values were calculated by fisher’s exact test.
